# Scepter dual‐lumen balloon catheter for Onyx embolization for dural arteriovenous fistula

**DOI:** 10.1186/s12883-021-02046-6

**Published:** 2021-01-20

**Authors:** Chang Ki Jang, Byung Moon Kim, Keun Young Park, Jae Whan Lee, Dong Joon Kim, Joonho Chung, Jun-Hwee Kim

**Affiliations:** 1 Departments of Neurosurgery, Severance Stroke Center, Severance Hospital, Yonsei University College of Medicine, Seoul, South Korea; 2 Division of Interventional Neuroradiology, Department of Radiology, Severance Stroke Center, Severance Hospital, Yonsei University College of Medicine, 50 Yonsei-ro, 120-752, Seodaemun-gu, Seoul, South Korea

**Keywords:** Dural arteriovenous fistula, Onyx embolization, Dual‐lumen balloon catheter

## Abstract

**Background:**

This study aimed to evaluate the efficacy and safety of Scepter dual-lumen balloon catheter for transarterial Onyx embolization of dural arteriovenous fistula (DAVF).

**Methods:**

Transarterial Onyx embolization using a Scepter dual-lumen balloon catheter (Scepter-assisted Onyx embolization) for DAVF was attempted in a total of 35 patients (mean age, 52.5 years; M:F = 24:11) between October 2012 and December 2018. The results of Scepter-assisted Onyx embolization were evaluated with respect to total procedural and Onyx injection times, the types and number of feeders requiring embolization, angiographic and clinical outcomes, and treatment-related complications.

**Results:**

Initial presentations were non-hemorrhagic neurological deficits in 10, intracranial hemorrhage in 8, seizure in 7, headache in 7, and intractable tinnitus in 3. All DAVF were aggressive type (Borden type 2, 14.3 %; type 3, 85.7 %). Scepter-assisted Onyx embolization resulted in immediately complete occlusion in 33 patients (94.3 %) and near complete occlusion in 2 patients. Middle meningeal artery (51.4 %) was the most commonly used for Scepter-assisted technique, followed by occipital artery (42.9 %), ascending pharyngeal artery (2.9 %) and superficial temporal artery (2.9 %). There was no difference in complete occlusion rate between middle meningeal artery and the other arteries (94.4 % versus 94.1 %). The median number of total feeders embolized was 1 (range, 1–3). The median total procedural time was 45 minutes (range, 21 minutes – 127 minutes) and the median Onyx injection time was 11 minutes (range, 3 minutes – 25 minutes). All patients recovered completely (n = 31) or partially (n = 4) from presenting symptoms. Treatment-related complications occurred in 2 patients, of whom one had a permanent morbidity (2.8 %, ipsilateral facial nerve palsy). No patient showed a recurrence on follow-up imaging (median, 15 months; range, 3–56 months).

**Conclusions:**

Scepter-assisted transarterial Onyx embolization showed a very high complete occlusion rate with a low morbidity and no recurrence in aggressive type DAVF. Scepter dual-lumen balloon catheter seems to be a useful tool for transarterial Onyx embolization of DAVF.

## Background

Multi-therapeutic options such as stereotactic radiosurgery, microsurgery and endovascular treatment are used for treatment of DAVF [1, 2]. The Onyx Liquid Embolic agent (eV3 Endovascular, Plymouth, Minnesota) facilitates controlled penetration into the fistula, thereby improving the cure rate of endovascular mean. Therefore, Onyx has been a popular agent for AVF and AVM. Effective penetration requires an antegrade flow of Onyx from the feeding artery to the fistula site. With conventional microcatheter-based techniques, an initial proximal plug is often necessary, unless the tip of a microcatheter could be positioned completely wedged at the target feeding artery. That is also true with a detachable microcatheter. Subsequently, conventional technique takes a considerable amount of time to form a firm plug in order to facilitate effective penetration and to avoid reflux of Onyx. Recently, several small case series have documented that use of a dual-lumen balloon catheter (Scepter, Microvention, Tustin, California) enables more effective penetration and requires shorter injection time than the conventional microcatheter technique [3, 4].

The objective of this study was to evaluate the efficacy and safety of Scepter dual-lumen balloon catheter for transarterial Onyx embolization of dAVF.

## Methods

The Institutional Review Boards at all participating hospitals involved approved this retrospective study and waived the patient informed consent for study inclusion. Written informed consent for embolization was obtained from the patient or a legal representative before treatment.

All patients in whom the Scepter dual-lumen balloon catheter was attempted as a primary delivery tool for Onyx embolization for aggressive-type DAVF were identified from a prospectively maintained neurointerventional database in 3 hospitals between October 2012 and December 2018. Aggressive-type was defined as DAVF with cortical reflux (Borden type 2) or a directly draining cortical vein (Borden type 3).

### Transarterial Onyx embolization

All procedures were performed under general anesthesia. If it had been one of the feeders of DAVF, middle meningeal artery was preferentially selected for Onyx embolization. For overcoming the difficulty, if any, in navigation of the Scepter balloon catheter, the following techniques were used. (1) The tip of scepter balloon catheter was almost always steam-shaped for improving navigability. (2) The distal access catheter has always been used combined with a Shuttle 6F sheath for more supporting the Scepter balloon catheter. (3) If needed, exchange technique was employed for placing the scepter balloon catheter into the target point of the feeding artery using a microcatheter with inner lumen of 0.0165-inch and a 300-cm length, 0.014-inch exchangeable microwire (Transend, Stryker). In detail of exchange technique, a 0.0165-inch microcatheter was navigated to the target point of the feeding artery. An exchangeable wire was placed followed and the microcatheter was removed. Finally, Scepter balloon catheter was advanced over the exchange wire. In addition, vasodilator (Nimodipine, 0.3–0.5 mg) was infused into the feeding artery before the navigation of Scepter catheter to prevent vasospasm. The essential prerequisite of the target position of the Scepter balloon catheter within the feeding artery is that there is no functioning arterial branch arising between the point and the fistula.

### Main Outcomes

We evaluated the results of Scepter-assisted transarterial Onyx embolization with respect to treatment-related complications, angiographic outcome, total procedural time, Onyx injection time, and number of feeders needed for completion of embolization. Angiographic outcomes were classified as complete occlusion, near complete occlusion, or incomplete occlusion. Complete occlusion of d-DAVF was defined as no visualization of the isolated sinus or shunted cortical vein in the delayed venous phase on the completion angiogram. Near complete occlusion was defined as delayed visualization of the isolated sinus or shunted vein on the venous phase, with marked stasis of contrast material (≥ 2 seconds). Incomplete occlusion was defined as visualization of the isolated sinus or shunted vein on the arterial phase of the completion angiogram, with stasis of contrast material < 2 seconds.

Routine follow-up imaging was scheduled at 6 months with magnetic resonance angiography (MRA) or digital subtraction angiogram (DSA) and 24 months after the embolization. MRA was used for first follow-up modality in 20 cases and DSA was in 15 cases. MRA was routinely used for second follow-up, unless patient’s presenting symptoms were not improved or aggravated. If MRA source images show any high signal on the lesion in cases that MRA was used for follow-up modality, or patient’s presenting symptoms were not improved or aggravated, DSA was performed for confirmation. Angiographic and follow-up imaging outcomes were assessed by 2 independent raters who did not participated in the treatment. Inter-rater agreements were almost perfect for both immediate and follow results (*kappa* value = 0.97 and 0.93, respectively).

### Statistical analysis

Since this study evaluated the efficacy and safety of Scepter balloon catheter as a primary tool for Onyx embolization of DAVF without comparison with the other tools or methods, only descriptive statistics are presented. All data are presented as a mean and range for continuous variables and as a number and percentage for categorical variables.

## Results

Of the 53 patients who underwent transarterial embolization for cranial dAVF between September 2012 and December 2018. The Scepter balloon catheter was attempted to navigate to the target point (distal to the origin of potentially dangerous branch) of the feeding artery in 36 patients. Of them, the navigation of Scepter balloon catheter failed in 1 patient and Scepter balloon catheter was used as a primary delivery tool for Onyx delivery for DAVF in a total of 35 patients (97.2 %; mean age, 52.5 years; M:F = 24:11). Baseline clinical and angiographic characteristics of the patients are summarized in Table 1. Initial presentations were non-hemorrhagic neurological deficits in 10, intracranial hemorrhage in 8, seizure in 7, headache in 7, and intractable tinnitus in 3. All DAVF were aggressive type; Borden type 2, 5 (14.3 %) and type 3, 30 (85.7 %), respectively.

**Table 1:** Baseline clinical and angiographic characteristics

Age, mean (range), years	52.5 (18–78)
Male, n (%)	24 (68.6)
Presentation, n (%)	
Non-hemorrhagic neurological deficits	10 (28.6)
Intracranial hemorrhage	8 (22.9)
Seizure	7 (20.0)
Intractable Headache	7 (20.0)
Intractable tinnitus	3 (8.5)
Location, n (%)	
Transverse-sigmoid sinus	13 (37.1)
Juxta-sinus lacuna (bridging vein between cortical veins and sinus)	13 (37.1)
Superior petrosal sinus	5 (14.3)
Torcula	3 (8.6)
Sphenoparietal sinus	1 (2.8)
Type, n (%)	
Borden type 2	5 (14.3)
Borden type 3	30 (85.7)

Procedural details, clinical and angiographic outcomes are summarized in Table 2.

**Table 2:** Procedural details, clinical and angiographic outcomes of Scepter-assisted Onyx embolization

Planned adjunctive NBCA embolization of another feeder(s) immediately before Onyx embolization, n (%)	2 (5.7)
Total number of feeders embolized, median (range)	1 (1–3)
Feeding artery selected for Scepter-assisted technique	
Middle meningeal artery, n (%)	18 (51.4)
Occipital artery, n (%)	15 (42.9)
Superficial temporal artery, n (%)	1 (2.9)
Ascending pharyngeal artery, n (%)	1 (2.9)
Total procedural time, mean ± SD (range), minutes	43 ± 28 (21–127)
Onyx injection time, mean ± SD (range), minutes Injection time through middle meningeal artery (n = 18) Injection time through occipital artery (n = 15)	11 ± 7 (3–25) 9 ± 4 (3–17)* 13 ± 9 (8–25)*
Immediate angiographic results, n (%)	
Complete occlusion	33 (94.3)
Near complete occlusion	2 (5.7)
Treatment-related permanent morbidity, n (%)	1 (2.8)
Clinical follow-up duration, median (range), months	25 (3–78)
Clinical follow-up results, n (%)	
Complete resolution of presenting symptoms	30 (85.7)
Partial improvement from presenting symptoms	5 (14.3)
Imaging follow-up duration, median (range), months	15 (3–56)
Imaging follow-up results (n = 33), n (%)	
Complete occlusion	32 (97.0)
Near complete occlusion	1 (3.0)

*Significantly different (*p < 0.05*)

**Fig. 1:**
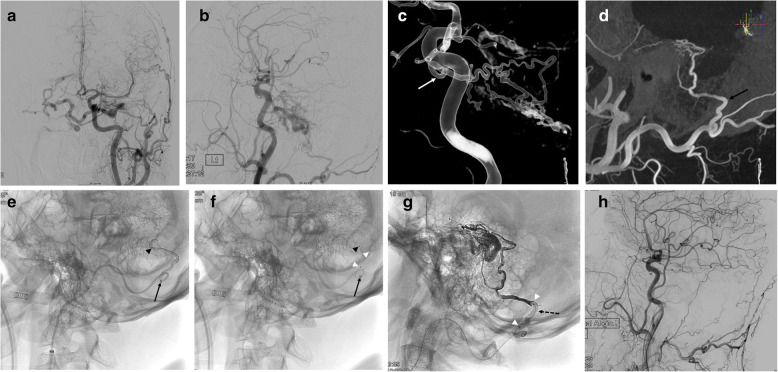
A 49-year-old man presenting with intracranial hemorrhage. **a** and **b **Anterior (**a**) and lateral (**b**) angiograms show dural arteriovenous fistula at the left superior petrosal sinus with reflux into contralateral medullary veins. **c** and **d** Transparent image (**c**) of 3D angiogram and thick slice maximum intensity reconstruction image (**d**) demonstrate two main feeding arteries, inferolateral trunk (white arrow) and occipital artery branch (black arrow). Note that inferolateral trunk has very tortuous course and occipital artery branch has trans-osseous course. **e** A microcatheter with inner diameter of 0.0165-inch is navigated into the occipital branch with a 0.014-inch wire. Arrowhead indicates the tip of microcatheter and arrow indicates 5-F intermediate catheter. **f** A Scepter-C dual lumen balloon catheter is placed using exchange technique with 0.014-inch 300-cm length exchangeable wire. Black arrowhead indicates the tip of Scepter-C and white arrowheads indicate proximal and distal markers of balloon. **g** A spot image near the end of the procedure. Dashed arrow indicates inflated balloon. **h** The final control angiogram at the common carotid artery reveals complete occlusion of the fistula

**Fig. 2:**
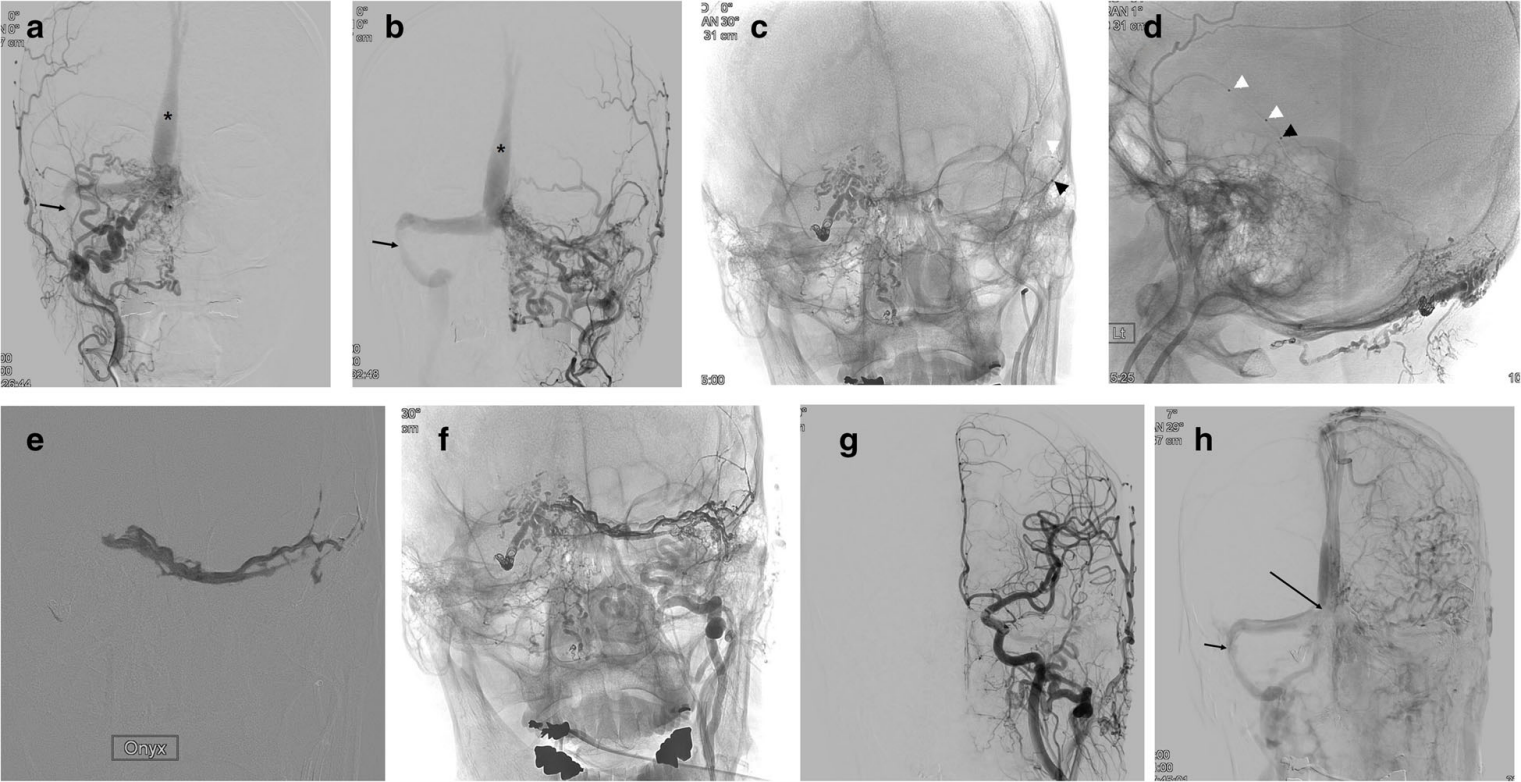
A 60-year-old man presenting with intractable tinnitus and dizziness. **a** and **b** Right (**a**) and left (**b**) external carotid artery angiograms reveal dural arteriovenous fistula at the torcula, which shows numerous feeding arteries and reflux to superior sagittal sinus (asterisk). Note that left transverse sinus is occluded and right transverses-sigmoid sinus shows focal stenosis (arrow). **c** and **d** Frontal (**c**) and lateral (**d**) spot images shows the position of Scepter-C dual lumen balloon catheter at the left middle meningeal artery. Note that proximal coil-protected embolizations were done using the mixture of N-butylacrylate and contrast material two times at the right occipital artery branches. Black arrowhead indicates the distal marker and white arrowheads indicate proximal and distal balloon markers of Scepter-C. **e** A spot image during the Onyx injection through the Scepter-C under roadmap. **f** and **g** Non-subtracted (**f**) and subtracted (**g**) angiograms reveal complte occlusion of dural arteriovenous fistula. **h** Venous phase angiogram after balloon angioplasty for the stenosis show patent torcula (long arrow) and improved stenosis of right transvers-sigmoid sinus

Scepter-assisted Onyx embolization resulted in immediately complete occlusion in 33 patients and near complete occlusion in 2 patients. Exchange technique was applied with a 0.014-inch thickness and 300-cm length wire (Transcend exchangeable wire, Stryker or Grandslam, Abbort, ) for navigation of Scepter dual-lumen balloon catheter to a target position in a very tortuous feeder artery in 5 patients (14.3 %).(Fig. 1) Planned adjunctive embolization with N-butyl cyanoacrylate was conducted in 2 patients (5.7 %) with multiple feeders immediately before Scepter-assisted Onyx embolization.(Fig. 2) The median number of total feeders embolized was 1 (range, 1–3). Middle meningeal artery (51.4 %) was the most commonly used for Scepter-assisted technique, followed by occipital artery (42.9 %), ascending pharyngeal artery (2.9 %) and superficial temporal artery (2.9 %). There was no difference in complete occlusion rate between middle meningeal artery and the other arteries (94.4 % versus 94.1 %). The median total procedural time was 45 minutes (range, 21 minutes – 127 minutes) minutes and the median Onyx injection time was 11 minutes (range, 3 minutes – 25 minutes). Comparing the Onyx injection time between middle meningeal artery and occipital artery, it was significantly shorter at the middle meningeal artery (9 ± 4 minutes) than at the occipital artery (13 ± 9 minutes). (Table 2) All patients recovered completely (n = 31) or partially (n = 4) from presenting symptoms. Treatment-related complications occurred in 2 patients, of whom one had a permanent morbidity (2.8 %, ipsilateral facial nerve palsy). One patient presented with intractable right facial spasm due to neurovascular compression from the engorged draining vein of the dAVF at right superior petrosal sinus. The patient experienced right facial palsy after Onyx embolization, likely due to penetration of Onyx into the petrous branch, which supplies the petrous segment of the facial nerve via artery-to-artery anastomosis. The other patient experienced a transient contralateral sensory change but completely recovered in a week.

No patient showed a recurrence on follow-up imaging (median, 15 months; range, 3 months – 56 months).

## Discussion

The results of this study demonstrated that (1) the navigation of Scepter balloon catheter to the target point (distal to the potentially dangerous branch) of the feeding artery was successful in 97.2 % of the attempted cases, (2) Scepter-assisted transarterial Onyx embolization showed a very high successful (94.3 % complete and 5.7 % near complete) occlusion rate with a low permanent morbidity (2.8 %), (3) Scepter-assisted technique was as effective using the other feeding arteries as middle meningeal artery, and finally, (4) the Onyx injection (median, 11 minutes; range, 3–37 minutes) and total procedural (median 35 minutes; range, 21–127 minutes) times were quite shorter than those of conventional technique in the literature, onyx injection time ranged 30minutes from 15 to 60 minutes [5]. Since Scepter dual lumen balloon microcatheter was introduced [3], several small case series showed that it might be a promising Onyx delivery tool for the treatment of dural AVF. In this study, we evaluated that Scepter balloon catheter can be used as a primary tool for Onyx embolization, to our best knowledge, in the largest population.

Traditionally, transarterial embolization of dAVF has been associated with a high failure rate and was considered palliative or adjunctive to transvenous embolization. In the last 10 years, however, there has been a paradigm shift toward transarterial embolization of dAVF. Since its introduction, Onyx has gained widespread popularity as a primary embolic agent for various vascular lesions. Due to its cohesive property, Onyx can allow for a more controlled injection and penetration into the target lesion. As a result, contemporary series have consistently demonstrated cure rates with transarterial embolization exceeding 70 % [6]. Nevertheless, to control the direction of the Onyx toward the target lesion with conventional technique, the formation of a proximal plug around the microcatheter tip should be preceded before antegrade propagation of Onyx in most cases. This, in turn, results in increased procedural and fluoroscopy times. Furthermore, excessive and unwanted reflux of Onyx into proximal segment of the feeding artery may not rarely occur, which leads to incomplete occlusion or failure of embolization. Such a phenomenon is more common in highly resistant and tortuous artery such as occipital artery than middle meningeal artery. Therefore, most investigators recommend middle meningeal artery as a primary conduit for Onyx delivery [7–9].

Recently, Kim et al. reported a small case series that compared the results of conventional microcatheter technique (n = 14) and Scepter-assisted technique (n = 16). That study demonstrated that utilization of Scepter-assisted technique clearly increased the immediate complete occlusion rate (86.7 % versus 35.7 %) and decreased total procedural time (62 ± 32 minutes versus 171 ± 88 minutes), Onyx injection time (10 ± 6 minutes versus 49 ± 32 minutes, and number of feeding arteries (1 versus 2) required for completion of embolization. The results of the present study also showed similar results to those of Scepter-assisted technique group, which can provide additional evidence to the usefulness of Scepter-assisted technique.

When recommending Scepter balloon catheter as a primary delivery tool for Onyx embolization, the navigability of the balloon catheter into the target feeding artery is a major huddle. In this study, for navigation of the Scepter balloon catheter to a very tortuous feeding artery, exchange technique was used in 14.3 % and distal access catheter in addition to shuttle sheath has been routinely used since its introduction. Subsequently, the navigation of Scepter balloon catheter was attempted in approximately two thirds of all cranial dAVF and was successful in all but one. In fact, the navigation of the Scepter balloon catheter to the target point of the feeding artery succeeded in all cases after using a distal access catheter.

Middle meningeal artery is the most common feeding artery of cranial dAVF and recommended as a primary conduit for Onyx delivery in literature [7–10]. However, middle meningeal artery is not always the feeder artery of dAVF and furthermore often trivially contributes as a feeder. Occipital artery is a second most common contributing artery but has usually very tortuous course and high resistance because it is trans-osteal artery. Therefore, the cure rate of Onyx embolization with conventional technique is lower using occipital artery than middle meningeal artery. It has been suggested as a major cause of transarterial Onyx embolization failure with conventional technique [10]. In the present study, middle meningeal artery and occipital artery was selected for Scepter-assisted technique in 51.4 % and 42.9 %, respectively. However, there was no difference in complete occlusion rate between middle meningeal artery and the other arteries. It is largely because the firm inflated balloon of Scepter catheter makes it much easier to penetrate via a highly resistant occipital artery into the fistula. On the other hand, the Onyx injection time was shorter at the middle meningeal artery than at the occipital artery. It suggests that middle meningeal artery is better than occipital artery even for Scepter-assisted Onyx embolization in terms of the injection time if both arteries are usable.

The complication associated with onyx embolization include catheter entrapment, cranial nerve damage and distal propagation of onyx [11–13]. In meta-analysis of trans arterial onyx embolization for the intracranial dural fistulas, procedure-related morbidity was 3 % (95 % CI: 1 %, 5 %; I2, 0 %) [14]. In this study, treatment-related complications occurred in 2 patients, of whom one had a permanent morbidity (2.8 %, ipsilateral facial nerve palsy). There was no arterial rupture due to balloon over-inflation. All treated feeding arteries were external carotid artery branches which have external elastic lamina and thicker media, compared to intradural cerebral arteries in which external elastic lamina is absent. Therefore, the branches of external carotid artery may be quite endurable in the condition of stretching by balloon over-inflation. Even if dural arterial rupture happens, in our opinion, it can be easily controlled by further injection of onyx for embolization of the ruptured artery because any functioning branch do not arise from the target point of the artery. The final concern is entrapment of the balloon catheter. In our experience, however, although some distance of Onyx reflux occurred around the inflated balloon, deflation of the balloon easily separated the catheter from the Onyx coating vessel wall and retrieval of the Scepter catheter is very easy, taking less than 10 seconds in all cases.

### Limitation

This study has several limitations inherent to retrospective analysis. This study did not compare Scepter-assisted technique with the other technique. However, the prevalence of dAVF requiring treatment is not so high in our country enough for randomized study. Furthermore, although all consecutive cases of Onyx embolization for dAVF were not treated by Scepter-assisted technique, it was already a frontline technique for transarterial Onyx embolization in our hospital because the preceding result of the technique had been very promising as previously reported [4]. Another limitation is that DSA was not used for the first follow-up imaging in all cases, which may miss a small slow-flow residual fistula. However, because the sensitivity of evaluating dAVF using MRA source image was very high [4, 15] and no patient showed recurrent symptom or recanalization on the follow-up after complete occlusion, the durability of Scepter-assisted technique may be reliable.

## Conclusions

Scepter-assisted trans arterial Onyx embolization showed a very high complete occlusion rate with a low morbidity and no recurrence in aggressive type DAVF. Scepter dual-lumen balloon catheter seems to be useful tool for transarterial Onyx embolization of DAVF.

## Data Availability

The datasets used and analyzed during the current study are available from the corresponding author on reasonable request.
